# Increased serum levels of sortilin-derived propeptide after electroconvulsive therapy in treatment-resistant depressed patients

**DOI:** 10.2147/NDT.S170165

**Published:** 2018-09-06

**Authors:** Morgane Roulot, Alessandra Minelli, Marco Bortolomasi, Elisabetta Maffioletti, Massimo Gennarelli, Marc Borsotto, Catherine Heurteaux, Jean Mazella

**Affiliations:** 1 Molecular and Cellular Institute of Pharmacology, CNRS, Institut de Pharmacologie Moléculaire et Cellulaire, UMR 7275, Université Côte d’Azur, Valbonne, France, mazella@ipmc.cnrs.fr; 2 Department of Molecular and Translational Medicine, Biology and Genetic Division, University of Brescia, Brescia, Italy; 3 Psychiatric Hospital “Villa Santa Chiara”, Verona, Italy; 4 Genetic Unit, IRCCS Istituto Centro San Giovanni di Dio Fatebenefratelli, Brescia, Italy

**Keywords:** sortilin propeptide, electroconvulsive therapy, treatment-resistant depressed patients, diagnosis

## Abstract

**Purpose:**

Sortilin-derived propeptide (PE) and its synthetic analog spadin show strong antidepressant activity in rodents and, therefore, could be used as a biomarker to evaluate the clinical efficacy of antidepressant treatments. The aim of this study was to determine whether electroconvulsive therapy (ECT) modulates serum PE concentration in patients with treatment-resistant depression (TRD).

**Patients and methods:**

Forty-five patients with major depressive disorder, who met the *Diagnostic and Statistical Manual of Mental Disorders-IV* criteria, were selected for this study.

**Results:**

We did not observe any difference in the PE levels between TRD patients and controls (*z*=0.10, *P*=0.92), but we found a strong significant increase between the PE levels measured just before (T0) and about 1 month (T2) after ECT (*z*=−2.82, *P*=0.005). A significant difference between T0 and T2 was observed only in responders (*z*=−2.59, *P*=0.01), whereas no effect was found in nonresponders (*z*=−1.27, *P*=0.20). Interestingly, we found a significant correlation between the increase in PE levels and decrease in Montgomery -Åsberg Depression Rating Scale scores for the total patient sample (*P*=0.03).

**Conclusion:**

This study indicates for the first time that ECT affects serum PE concentration in responders and, therefore, could contribute to the evaluation of the therapy success.

## Introduction

The inefficacy of pharmacological treatments for patients with major depressive disorder (MDD) may reach up to 30% and remains as a crucial problem for psychiatrists and patient’s family. The resulting drug-resistant population corresponds to “treatment-resistant depression” (TRD).^1^,^2^ However, several trials have been successfully performed to ameliorate antidepressant (AD) efficacy by adding vitamins or statins.^3^,^4^ To date, several alternative treatments are available, such as electroconvulsive therapy (ECT) and nonpharmacological treatments like cognitive behavior therapy or interpersonal psychotherapy.^5^ ECT is used to induce neuromodulation,^6^ which is highly effective in TRD with 60%–80% of patients achieving remission.^7^,^8^ Interestingly, combination of ECT with aerobic exercise training ameliorates the remission rates of patients with MDD when compared with ECT alone.^9^ Although the mechanisms of action of ECT remain unknown, this technique alters the levels of several brain molecules including neurotransmitters, neuropeptides, and neurotrophic factors.^10^,^11^ In the periphery, ECT also modifies the serum levels of proteins and small molecules including sortilin,^12^ brain-derived neurotrophic factor (BDNF),^13^,^14^ and VEGF.^15^

Sortilin, known to control the intracellular sorting of BDNF to the regulated secretory pathway,^16^ is a multifunctional protein expressed both in the central nervous system and in the periphery^17^ as a proprotein whose maturation leads to the release of a propeptide (PE) of 44 amino acids.^18^ We recently demonstrated that PE and its shorter synthetic analog spadin display potent AD action in rodents.^19^ The AD activities of PE and spadin were studied by inhibiting the potassium channel TREK-1 activity,^19^ which is a target for depression.^20^ More recently, we developed a method that allowed us to selectively measure and compare the sortilin-derived PE concentrations in the serum of healthy controls and of MDD patients before and after AD treatment.^21^ We observed that the PE levels were significantly decreased in the serum of MDD patients. Interestingly, these levels were restored to the levels of healthy nonpsychiatric controls after pharmacological AD treatment. These observations led us to postulate that the longitudinal quantification of serum PE concentrations could assist psychiatrists in evaluating the efficacy of AD response. For these reasons, we addressed here the possibility that the sortilin-derived PE can be used as a biological marker to monitor the effectiveness of ECT in TRD patients. We measured PE concentrations in a cohort of TRD patients before and 1 month after ECT and observed that the PE levels significantly increased only in the serum of patients who responded to ECT.

## Materials and methods

### Sample

The control sample consisted of 49 unrelated healthy volunteers who were screened for *Diagnostic and Statistical Manual of Mental Disorders-IV* (DSM-IV) Axis I disorders by expert psychologists using the Mini-International Neuropsychiatric Interview. Only healthy volunteers without history of drug, alcohol abuse, or dependence and without a personal or first-degree family history of psychiatric disorders were enrolled in this study.

Forty-five treatment-resistant MDD (TRD) patients with severe depression, who met the DSM-IV criteria, were selected for this study. Unipolar depression was diagnosed using the Structured Clinical Interview for DSM-IV Axis I Disorders diagnostic scale. The exclusion criteria were as follows: 1) mental retardation or cognitive disorder; 2) a lifetime history of schizophrenic, schizoaffective, or bipolar disorder; 3) personality disorder, substance abuse, alcohol abuse or dependency, obsessive compulsive disorder, or posttraumatic stress disorder as the primary diagnosis; and 4) comorbidity with an eating disorder.

Furthermore, absence of relevant neurological diseases such as epilepsy and Parkinson’s syndrome in both patients and controls was a mandatory criterion for them to be included in the study. Finally, subjects who scored lower than 27/30 at the Mini Mental State Examination were excluded from the study.

Treatment resistance to ADs was defined as failure of a patient to respond to two or more adequate trials of two or more different AD classes including an adequate trial of a tricyclic antidepressant drug, referred to Stage III of Thase and Rush Staging Method.^22^ All patients were referred to the “Villa Santa Chiara” Psychiatric Hospital in Verona and scheduled to undergo ECT.

Among the 45 TRD patients, 33 (73.3%) showed psychotic symptoms; 10 (22.2%) showed current comorbidity of Axis I disorders (generalized anxiety disorder, panic attacks, panic disorders, or anxiety disorder not otherwise specified); and 11 (24.4%) showed symptoms of Axis II disorders (dependent or obsessive–compulsive personality disorder).

Illness severity and ECT outcome were assessed using the Montgomery–Åsberg Depression Rating Scale (MADRS) before the treatment (T0), the day after the last session of ECT (T1), and 1 month after ECT (T2). Treatment was given three times a week. The mean number of sessions received was 7.49±2.42, and ECT was completed based on the clinical judgment of the treating physicians. During the whole period of ECT and in the month after ECT, the pharmacological treatment was maintained stable, with only a possible slight reduction in dosage.

All the sociodemographical, clinical, and pharmacological treatment characteristics of the patients are shown in Table 1. For both patients and controls, venous blood samples were collected between 8:00 am and 9:00 am after an overnight fast in anticoagulant-free tubes. Blood samples were collected at the same time points (T0, T1, and T2) for the patients, whereas for the controls at the time of enrolment. Serum was separated by centrifugation (1,620× *g* for 15 minutes).

This study was approved by the local ethics committees (CEIOC IRCCS Istituto Centro San Giovanni di Dio Fatebenefratelli, Brescia N: 50/2008 and Ethics Committee of the Province of Verona N: 4997/09.11.01), and written informed consent was obtained from all participants.

### Electroconvulsive therapy

A medical history and a physical examination along with routine blood and urine examinations, an electrocardiogram, a cerebral computed tomography scan, and a chest film were requested to screen for general medical conditions. Anesthesia for ECT was routinely induced by intravenous thiopental sodium (3.0 mg/kg for males and 2.5 mg/kg for females). Muscle relaxation was achieved with intravenous succinylcholine (0.7 mg/kg). In addition, the patients were premedicated with atropine sulfate (0.5 mg intravenously).

ECT was performed between 7:00 am and 9:00 am three times per week using Thymatron^®^ DG (Somatics, Inc, Lake Bluff, IL, USA) with standard settings with a bipolar brief pulse square wave and bilateral electrode placement.^23^ The maximum ECT stimulus was a charge of 504 mC, with a current of 0.9 A, a frequency of 30–70 Hz, a pulse width of 1 ms, and a maximum duration of 8 seconds. Stimulus intensity was selected based on the age of the patients. The ECT was complete based on the clinical judgment of the treating physicians.

### Determination of serum PE concentration

The serum sortilin-derived PE concentration was determined in triplicate for each blood sample using AlphaLisa™ technology (Perkin Elmer). Briefly, the concentration of PE in the serum sample was determined from its percentage of signal inhibition between donor and acceptor beads linked in the presence of biotinylated spadin as previously described.^21^

### Statistics

Demographic and clinical characteristics were described either in quantitative term of mean±SD or as proportions. Parametric and nonparametric tests were used to meet relative assumptions (eg, distribution and sample size). Clinical changes that occurred during ECT were measured by MADRS and analyzed using a generalized linear model in a repeated measure design with time (T0, T1, T2) as a within-subject factor, and then the Greenhouse–Geisser correction was applied. Biological differences in PE levels were analyzed using the Wilcoxon signed rank test.

All statistical analyses were conducted using SPSS version 17.0 (SPSS Inc., Chicago, IL, USA).

## Results

**Figure 1:**
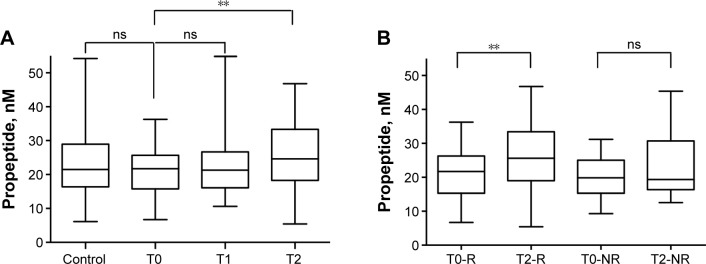
PE concentrations in sera from healthy controls and TRD patients. (**A**) PE concentrations in sera from healthy controls and TRD patients before (T0) or 1 day (T1) or 1 month (T2) after ECT. Statistical analysis was performed using Mann–Whitney *U* test between patients (TRD T0, n=45) and controls (n=49) and using Wilcoxon signed rank test between untreated (T0) and ECT-treated (T1 and T2) TRD. **P<0.01. (**B**) PE concentrations in sera from responders (R) and nonresponder (NR) TRD patients before (T0) and 1 month after ECT. **P<0.01. **Abbreviations:** TRD, treatment-resistant depression; PE, propeptide; ECT, electroconvulsive therapy; ns, nonsignificant.

We did not observe any difference in the PE levels for TRD patients and controls (*z*=0.10, *P*=0.92; Figure 1A), but we found a strong significant increase between the PE levels measured just before (T0) and 1 month (T2) after ECT (*z*=−2.82, *P*=0.005; Figure 1A). Comparison of PE levels measured at the baseline (T0) and just after ECT (T1) did not show any change (*z*=−0.67, *P*=0.50). It is important to underline that the significant difference in PE concentration between T0 and T2 was observed only in responder patients (*z*=−2.59, *P*=0.01), whereas in nonresponders, no difference was found (*z*=−1.27, *P*=0.20; Figure 1B).

**Figure 2:**
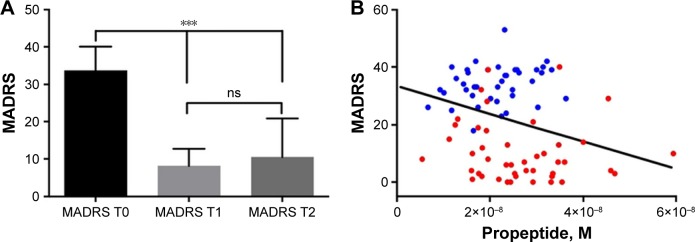
MADRS score evaluation of TRD patients. (**A**) Mean±SD of MADRS for TRD patients before (T0) or 1 day (T1) or 1 month (T2) after ECT. ***P<0.001. (**B**) A significant correlation was obtained between the concentration of PE (at T0 in blue and T2 in red) and their corresponding MADRS scores (*r*=−0.235, *P*=0.03). **Abbreviations:** TRD, treatment-resistant depression; MADRS, Montgomery–Åsberg Depression Rating Scale; ns, nonsignificant; PE, propeptide.

ECT reduced depression symptomatology as measured by MADRS (T0=33.67±6.41, T1=8.16±4.52, T2=10.47±10.38, *F*_2,88_=150.31, *P*<1.8 × 10^−22^; Figure 2A) by 69% on the MADRS total score. In particular, pairwise comparison indicated a significant decrease in MADRS scores between T0 and T1 (*P*<6.7 × 10^−26^) and between T0 and T2 (*P*<7.0 × 10^−15^). But no difference was observed between T1 and T2 (*P*=0.56). All *P*-values of pairwise comparison were already adjusted by Bonferroni correction. Interestingly, a significant correlation was obtained between the concentration of PE (at T0 and T2) and the corresponding MADRS scores (*r*=−0.235, *P*=0.03; Figure 2B). However, we did not observe any correlation with regard to changes in PE concentrations after ECT (*P*=0.5).

We observed a significant difference in age and education levels in controls compared to TRD patients, ie, differences that could alter our results on serum PE concentration (Table 1). However, we did not obtain significant correlations between education levels and PE concentrations in controls (*r*=−0.31, *P*=0.369) or in TRD patients (*r*=0.251, *P*=0.114). Similarly, no correlation was found between age and PE concentrations both in controls (*r*=0.213, *P*=0.165) and in patients (*r*=0.146, *P*=0.36), indicating that neither the age nor the education level may affect serum PE concentration. Interestingly, significant correlations were found between the % of difference in PE and age (*r*=0.38, *P*=0.035) and significant differences were observed between smokers and nonsmokers (*z*=−3.17, *P*=0.001). A significant difference in T0–T2 persisted only in nonsmokers (n=29; *z*=−3.54, *P*=0.0004) and not in smokers (n=16; *z*=−0.36, *P*=0.72).

## Discussion

The findings of this work clearly indicated that the TRD patients who underwent ECT had significantly higher serum PE concentrations compared with the values measured before therapy. The important point is that only responders (MADRS score reduced by more than 50%) showed a significant difference in serum PE levels between T0 and T2 (77.8% of patients), which indicates that the quantification of PE concentrations could be used to evaluate the response efficacy after treatment like ECT. Unchanged PE levels on the day following ECT, a time point where clinical effects are obvious, are not exceptional. Indeed, previous studies have shown unchanged levels of other biomarkers such as BDNF^13^,^24^ and VEGF^15^ that were measured between one and a few days after ECT. It is conceivable that a certain lapse of time is required to produce changes in PE levels, along with the therapeutic effectiveness.

It is to note that the PE levels in the serum of TRD patients before ECT were not significantly different from those of control healthy subjects (25.4±2.4 nM for TRD patients at T0 and 23.7±1.5 nM in controls). This appears to be contradictory to the results that were previously published on MDD patients with significantly lower serum PE concentrations compared with controls (18.9±1.3 nM).^21^ The discrepancy observed between the serum concentration of PE in MDD and TRD patients suggests that succession of several types of treatments increases the PE level even without remission and that further ECT reveals the possibility to use PE concentration as a marker for remission of the disease. Another explanation for the high PE level in TRD is that repetition of treatments may trigger a downregulation of PE targets leading to a compensatory increase of the serum PE concentration.

Serum PE concentration may be used as a specific biomarker in addition to sortilin, BDNF, and VEGF to validate the remission of depression after ECT in TRD patients. Indeed, ECT has already been described to increase the serum content of several polypeptides including BDNF^13^ and VEGF,^15^,^25^ as well as PE, that are decreased in MDD patients. The roles of VEGF, BDNF, and PE as actors in the mechanism of action of ECT or as consequences from other biological parameters remain to be determined. The effects of BDNF and PE in neurogenesis show their involvement in the remission of the pathology. Indeed, the release of both peptides depends on sortilin trafficking^16^,^18^ and the level of sortilin is also increased after ECT.^12^ The mechanism of action of PE and particularly its synthetic analog spadin has previously been characterized by the ability of the peptide to increase the neuronal membrane potential by blocking the TREK-1 potassium channels and to activate both the MAPK and PI3K signaling pathways.^26^ Also, spadin enhances, in vivo and in vitro, the expression of two markers of synaptogenesis, the postsynaptic density protein of 95 kDalton (PSD-95) and synapsin, resulting in the increase of mature spines in cortical neurons.^26^ Injections of spadin in mice have shown to increase BDNF in the hippocampus, defining the antidepressant action of the peptide.^26^ From these observations and from the existing observations which show that circulating PE varies according to the mood, we hypothesized that PE may function as a mood-enhancing hormone. It would be interesting to measure serum PE concentration during the period of remission in further experiments, in this case 1 month, to evaluate whether PE level could be increased prior to amelioration of the MADRS score. This could address an important point that there is a possibility of considering PE concentration as an earlier predictor of remission compared with MADRS score. The correlation between PE level and age and the differences between smokers and nonsmokers show that the PE concentration recovered in the serum likely changes as a function of age and/or of general physiological state. Further studies are necessary to assess this hypothesis.

## Conclusion

In conclusion, the serum PE concentration could be considered as a biomarker to evaluate both depression state^21^ and the remission of the disease after either a pharmacological or a noninvasive or invasive treatment. Further independent studies are required to confirm this hypothesis. In addition, it is likely that further use of PE analog spadin as a potent therapy against the pathology, a peptide for which the serum level could also be controlled, will definitely enhance the perspectives of new strategies to efficiently manage depression in the world.

## Figures and Tables

**Table 1:** Demographic and clinical characteristics of control and TRD patient groups as well as responders and nonresponders subgroups

Characteristics	Controls (N=49)	Total (N=45)	NR^a^ (N=10)	R^a^ (N=35)	*P*-value controls vs TRD
Age (years), mean (SD)	45.1 (12.1)	54.2 (14.0)	57.8 (11.8)	53.2 (14.6)	0.0001
Gender (%, F)	65.3	71.1	80.0	68.6	
Education (years), mean (SD)	13.8 (5.1)	7.9 (3.3)	8.1 (2.9)	7.9 (3.4)	<0.0001
BMI (body mass index)	23.6 (3.1)	26.6 (5.1)	26.3 (5.2)	26.7 (5.2)	0.0001
Smokers (%)	16.3	35.6	40.0	34.3	
Age of onset (years), mean (SD)		38.3 (14.7)	36.9 (13.9)	38.7 (15.1)	
MADRS (%) at T0, mean (SD)		33.7 (6.4)	30.8 (6.7)	34.5 (6.2)	
ΔMADRS (%) at T2, mean (SD)		−66.7 (33.4)	−12.2 (20.2)	−82.2 (14.6)	
Recurrent MDD (%)		93.3	100.0	91.4	
Severe vs moderate MDD (%)		100.0	100.0	100.0	
Psychotic symptoms (%)		73.3	70.0	74.3	
Comorbidity with personality disorders (%)		24.4	30.0	22.9	
Comorbidity with anxiety disorders (%)		22.2	40.0	17.1	
Comorbidity with alcohol abuse (%)		0.0	0.0	0.0	
Administration of antipsychotics^b^ (%)		77.8	80.0	77.1	
Administration of SSRIs^b^ (%)		60.0	40.0	65.7	
Administration of SNRIs^b^ (%)		26.7	20.0	28.6	
Administration of TCAs^b^ (%)		42.2	50.0	40.0	
Administration of NaSSAs^b^ (%)		28.9	20.0	31.4	
Administration of mood stabilizers^b^ (%)		8.9	0.0	11.4	

**Notes:**

a Response evaluated at 1 month after the electroconvulsive therapy (ECT). Patients were defined as responders if the percentage of MADRS reduction at T2 was >50%.

b The total number could exceed the number of subjects due to the presence of multiple drugs administration.

**Abbreviations:** R, responders; NR, nonresponders; SSRIs, selective serotonin reuptake inhibitors; SNRIs, serotonin–norepinephrine reuptake inhibitors; TCAs, tricyclic antidepressants; NaSSAs, noradrenergic and specific serotonergic antidepressants; MDD, major depressive disorder; MADRS, Montgomery–Åsberg Depression Rating Scale; TRD, treatment-resistant depression; T0, before ECT; T2, 1 month after ECT; ECT, electroconvulsive therapy.
